# Genome mining of *Escherichia coli* WG5D from drinking water source: unraveling antibiotic resistance genes, virulence factors, and pathogenicity

**DOI:** 10.1186/s12864-024-10110-x

**Published:** 2024-03-08

**Authors:** Oluwaseyi Samuel Olanrewaju, Lesego G Molale-Tom, Rinaldo K Kritzinger, Cornelius Carlos Bezuidenhout

**Affiliations:** Unit for Environmental Sciences and Management, North-West University, Potchefstroom Campus, Private Bag X6001, 2520 Potchefstroom, South Africa

**Keywords:** Comparative genomics, *Escherichia coli*, Genomic islands, Pathogenicity, Mobile genetic elements, Virulence factors

## Abstract

**Background:**

*Escherichia coli*, a ubiquitous inhabitant of the gut microbiota, has been recognized as an indicator of fecal contamination and a potential reservoir for antibiotic resistance genes. Its prevalence in drinking water sources raises concerns about the potential dissemination of antibiotic resistance within aquatic ecosystems and the subsequent impact on public health. The ability of *E. coli* to acquire and transfer resistance genes, coupled with the constant exposure to low levels of antibiotics in the environment, underscores the need for comprehensive surveillance and rigorous antimicrobial stewardship strategies to safeguard the quality and safety of drinking water supplies, ultimately mitigating the escalation of antibiotic resistance and its implications for human well-being.

**Methods:**

WG5D strain, isolated from a drinking water distribution source in North-West Province, South Africa, underwent genomic analysis following isolation on nutrient agar, anaerobic cultivation, and DNA extraction. Paired-end Illumina sequencing with a Nextera XT Library Preparation kit was performed. The assembly, annotation, and subsequent genomic analyses, including phylogenetic analysis using TYGS, pairwise comparisons, and determination of genes related to antimicrobial resistance and virulence, were carried out following standard protocols and tools, ensuring comprehensive insights into the strain’s genomic features.

**Results:**

This study explores the notable characteristics of *E. coli* strain WG5D. This strain stands out because it possesses multiple antibiotic resistance genes, encompassing tetracycline, cephalosporin, vancomycin, and aminoglycoside resistances. Additionally, virulence-associated genes indicate potential heightened pathogenicity, complemented by the identification of mobile genetic elements that underscore its adaptability. The intriguing possibility of bacteriophage involvement and factors contributing to pathogenicity further enriches our understanding. We identified *E. coli* WG5D as a potential human pathogen associated with a drinking water source in South Africa. The analysis provided several antibiotic resistance-associated genes/mutations and mobile genetic elements. It further identified WG5D as a potential human pathogen. The occurrence of *E. coli* WG5D raised the awareness of the potential pathogens and the carrying of antibiotic resistance in drinking water.

**Conclusions:**

The findings of this study have highlighted the advantages of the genomic approach in identifying the bacterial species and antibiotic resistance genes of *E. coli* and its potential as a human pathogen.

**Supplementary Information:**

The online version contains supplementary material available at 10.1186/s12864-024-10110-x.

## Background

Safe drinking water is crucial for public health, as contaminated water can lead to various waterborne diseases, including diarrhea [1]. To improve the microbial quality of drinking water, interventions such as filtration, disinfection, and water safety plans have proven effective [2]. Furthermore, water treatment plants play a vital role in ensuring the safety of drinking water, comprising different units, such as sedimentation, coagulation, filtration, and disinfection, which work together to remove contaminants and pathogens from the water [3, 4]. However, the effectiveness of water treatment plants in preventing waterborne diseases can be compromised if there are inadequate microbial barriers or if the treatment process is not properly managed [5]. One specific concern in water treatment plants is the presence of antimicrobial resistance genes (ARGs). These genes can pose a public health risk as they contribute to the spread of antibiotic resistance. A study conducted in China reported the presence of ARGs in both influent and effluent water samples from sand-settling reservoirs and drinking water treatment plants [6]. This highlights the importance of monitoring and managing the presence of ARGs in water treatment processes to minimize the risk to public health.

Drug-resistant *Escherichia coli* (*E. coli*) has emerged as a major public health concern due to the increasing prevalence of antimicrobial resistance (AMR) [7] and its role as a causative agent of various infections [8–10]. Multidrug-resistant *E. coli* strains have been detected in diverse environments, posing risks to both human and animal health [11–14]. Reservoirs of AMR *E. coli* have been identified in poultry farms, soil, surface water, and animal intestinal tracts [9, 15, 16]. Additionally, *E. coli* is a frequent cause of urinary tract infections (UTIs) among women of reproductive age [17, 18], while pregnant women are particularly vulnerable to UTIs [19]. Although commensal *E. coli* strains in the intestinal tract are generally non-pathogenic [20, 21], the presence of certain virulence genes may indicate an increased risk of pathogenicity. Moreover, recent research has challenged the assumption that *E. coli* solely indicates fecal contamination in drinking water, suggesting that it can grow in the environment independently of fecal sources [22].

Leveraging genome mining techniques to elucidate the presence of secondary metabolite gene clusters associated with antimicrobial resistance and virulence factors can provide critical insights into the underlying genetic determinants of *E. coli*’s pathogenicity and inevitably potential therapeutic applications. Hence, this study aims to employ genome mining to comprehensively explore the genetic landscape of the isolated *E. coli* strain, focusing on antibiotic resistance genes, virulence factors, and pathogenicity-related determinants. The findings from this investigation will contribute to the understanding of AMR in *E. coli* and shed light on the factors influencing its pathogenic potential, ultimately guiding future strategies for combating *E. coli*-related infections and improving public health outcomes.

## Results

### Genome properties

**Fig. 1:**
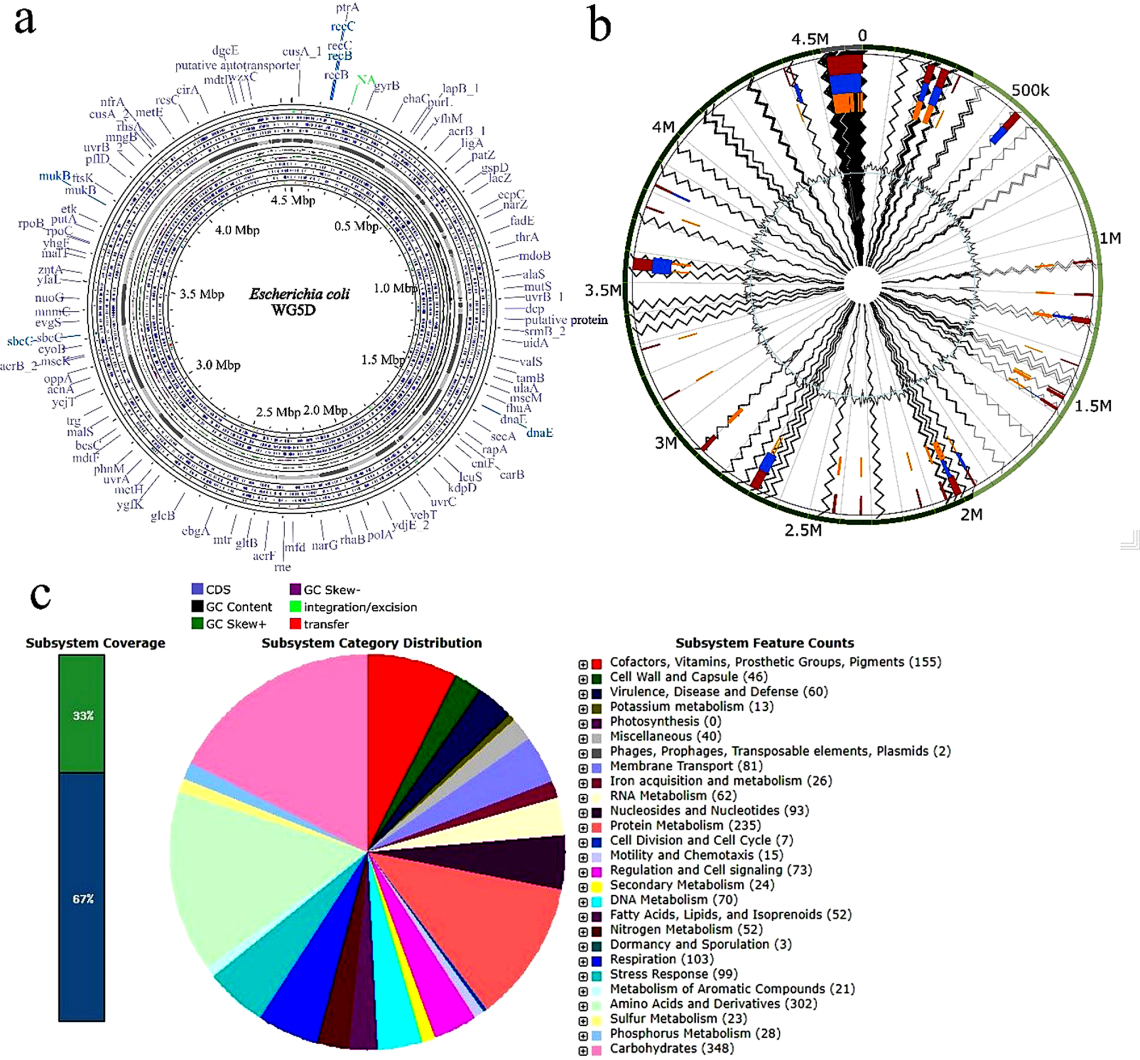
Genome properties of *E. coli* WG5D. (**a**) Circular visualization of *E. coli* strain WG5D genome (**b**) Circular plot of the genomic islands (GIs) identified in the strain WG5D chromosome. The orange bars represent the predicted GIs identified by SIGI-HMM, the blue bars represent the analysis by IslandPath-DIMOB, and the red boxes represent the integrated search results (**c**) Analysis of the protein-encoding genes (PEGs) assigned to subsystems categories according to the RAST server. The bar on the left presents the percentage of PEGs assigned to subsystems (green) and the PEGs that could not be placed into any subsystem (blue). The pie chart in the center depicts the subsystem category distribution. The colored categories on the right indicate the subsystem feature counts

WG5D genome was categorized as belonging to *Escherichia coli* based on the result on the GTDB (Table S1). The finally assembled *E. coli* WG5D genome consisted of 119 contigs with a total genome size of 4,538,266 bp and a GC content of 51.0% based on RAST annotation (Fig. 1a). The N50 size and L50 size were equal to 131,196 bp and 12, respectively. A total of 4429 protein-coding sequences (CDSs) and 92 total RNA were found in the genome. A total of 39 genome islands (GIs) were identified by the IslandViewer (Fig. 1b, Table S2), while the RAST database subsequently categorized the subsystem distributions of the genome into 369 categories (Fig. 1c, Table S3).

In addition, 39 GIs encoding various genes were identified in the *E. coli* WG5D genome (Fig. 1b, Table S2). The functions of some of the identified encoded genes in the GIs include stress resistance, VOC production, and antimicrobial resistance. However, many of the GI functions are unknown. These results suggest that the genes from GIs probably have a horizontal origin from another bacterial genus. Some of the identified islands encoded genes include transcriptional regulators, synthases (*YfjR, YkgA, RclR, AllS, YdeO, YeeN, EvgA, CsiR, PcoR, YjgJ, YagL, MraZ, YebC*), secretion systems (T6SS, T3SS, *YscJ*, *HrcJ*, *EscJ*, *PscJ*, *EprH*), insertion sequence elements, metal resistance and transport systems (*CopCDG*, *CusABCFRS*, *silE*, *PcoE*), multidrug efflux system (*EmrKY-TolC*), phage proteins (*YbcV*, *YdfU*, *cll*, *cro*), and toxin related proteins (*Ykfl*, *YafW, RelB, HigB, YeeU, YeeV, RatA*), among others (Table S2). Two phage regions harboring 30 phage genes were identified (Fig. S1) but no CAZymes and CRISPR elements were predicted.

In addition, the RAST server subsystem and non-sub-system coverage were 33% and 67%, respectively (Fig. 1c). The top three subsystem distributions were carbohydrates, amino acids and derivatives, as well as protein metabolism with 348, 302, and 245 genes respectively (Fig. 1c). RAST-based functional annotation identified the various genes associated with virulence, disease, and defense, membrane transport, iron acquisition and metabolism, and flagellar biosynthesis (Table S3). WG5D genome possessed genes for virulence disease and defense *viz. a viz.* genes for adhesion (*YidQRS* genes), resistance to antibiotics and toxic compounds (*CopCDG*, *CueO*, *CusRS*, *CutACEF*, *CorC*, and *ZitB* genes), and intracellular resistance (*Translation elongation factors G, Tu*, *Quinolinate synthetase*, and *Translation initiation factor 3*). In addition, fluoroquinolone resistance-associated genes such as *DNA gyrase* A and B were identified (Table S3). Among those implicated in membrane transport, we observed genes for the type II secretion system, type VII (*StfACDEFG*, and *CFA/I*), type VIII (*CsgEDCAFG*), and the type IV protein secretion system (*PilBQNOCPTAM*). In contrast, the observed genes for flagellar biosynthesis include the flagellar motor switch proteins (*FliMN*), flagellar ring protein for the structure (*FlgH*), flagellar biosynthesis proteins (*FlhBRA*), flagellar rotation proteins (*MotAB*), flagellar basal body rod modification protein (*FlgD*). Similarly, siderophore-associated genes such as F*epBCDEG*, *EntBHS*, (for biosynthesis of enterobactin siderophore) and *FhuABCD* (for aerobactin siderophore biosynthesis). Furthermore, various stress-tolerant genes (*Aquaporin Z*, *OsmY*, *YehYWZX*, *BetT*), Glucan biosynthesis proteins, *Choline dehydrogenase*, and Glycerol uptake facilitator protein were all identified for osmotic stress tolerance, while *Superoxide dismutase*, *Cytochrome c551 peroxidase*, *SoxR*, *FUR*, *NsrR*, *Glutathione synthetase*, *YncG*, *YghU*, YfcFG, *Glutathione peroxidase*, and *Glutaredoxin* 1/2/3 were identified for oxidative stress tolerance. In addition, *GadE*, *HdeDAB*, *RseAB*, *DegSQ*, *RasP*/*YluC*, and *HtrA* proteins were identified for periplasmic stress tolerance. Two phage and prophage biosynthesis genes (*IbrB* and *IbrA*) were also identified in the genome annotation. Other genes identified include 3 genes for dormancy and sporulation and 5 genes for iron acquisition and metabolism, among others (Table S3).

### Genome-based phylogenetic analysis

**Fig. 2:**
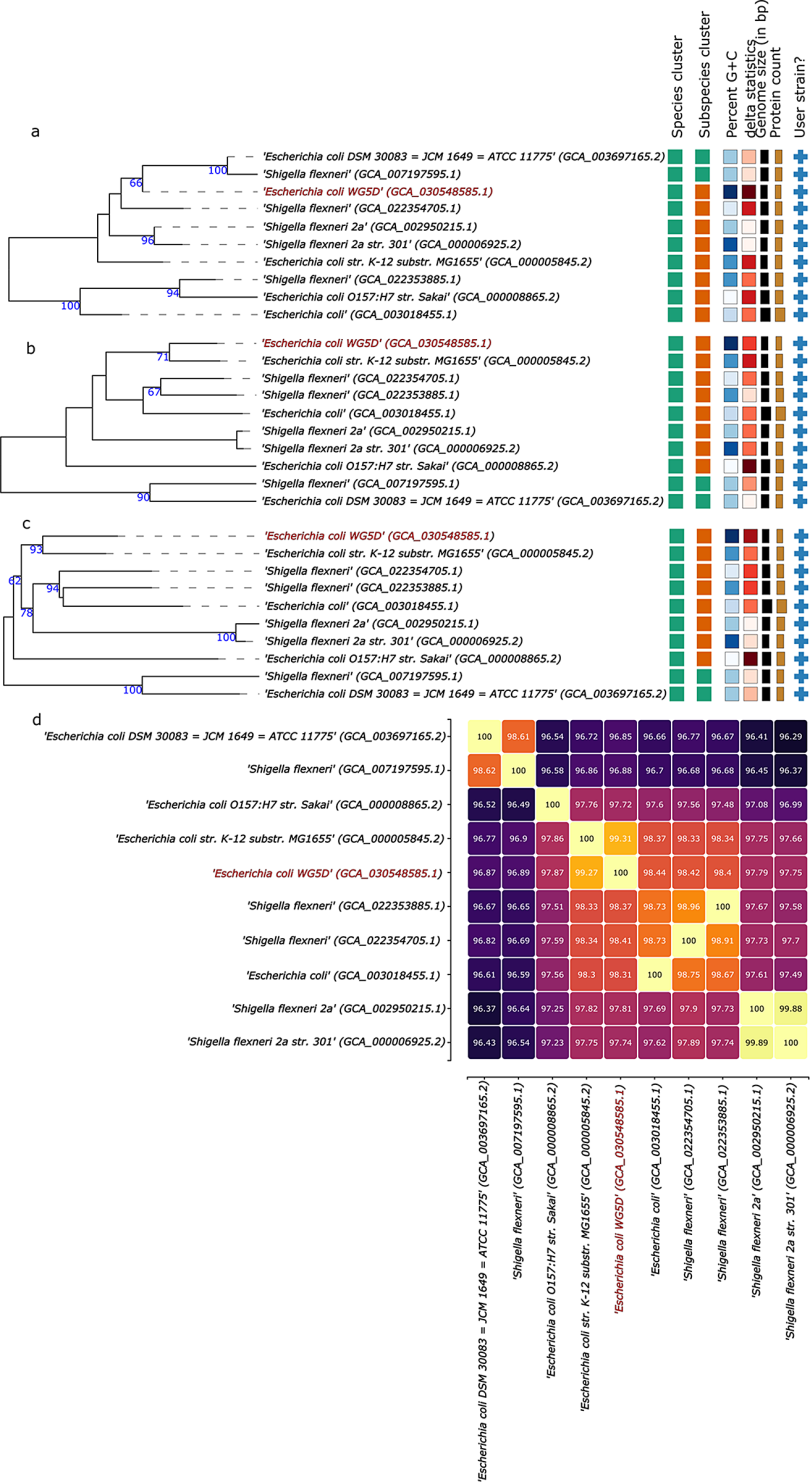
Tree inferred with FastME 2.1.6.1 from GBDP distances calculated from (**a**) 16 S rDNA gene sequences, (**b**) genome-based phylogeny. (**c**) proteome-based phylogeny. The branch lengths are scaled in terms of GBDP distance formula d5. The numbers above branches are GBDP pseudo-bootstrap support values > 60% from 100 replications, with an average branch support of 70.4%. The tree was rooted at the midpoint. (**d**) ANI demonstrating nucleotide-level genomic similarity

Taxonomic and functional research on microorganisms has increasingly relied on genome-based data and techniques [23]. Phylogenetic analysis based on whole-genome sequencing data is a powerful tool for studying the evolution and epidemiology of bacterial species or lineages [24]. The results of the 16 S rRNA sequence-based phylogenetic analysis (Fig. 2a), the genome-based phylogenetic analysis (Fig. 2b), and proteome-based phylogenetic analysis (Fig. 2c) showed that WG5D belongs to *E. coli*. DNA-DNA hybridization (DDH) and average nucleotide identity (ANI) have emerged as important for prokaryotic species circumscriptions at the genomic level [25]. Genome-genome distance calculator (GGDC), which mimics the DDH, was used to calculate the genome distances among the species. In contrast to the proposed threshold of 95% for the bacterial species delineation [25], the ANI values between the strain WG5D and the selected species ranged from 96.29 to 99.88%. The results of the 16 S rRNA sequence-based phylogenetic analysis (Fig. 2a), the genome-based phylogenetic analysis (Fig. 2b), and the proteome-based phylogenetic analysis (Fig. 2c) agreed on the same conclusions that WG5D belongs to *Escherichia coli*.This was further confirmed by the ANI analysis ( Fig. 2d) which indicated that strain WG5D is closely related to *E. coli* k12 with ANI value of 99.21%. In addition, it is not unusual to see that strain WG5D is also close to *Shigella* species because *Shigella* species and *E. coli* species are very similar, and genetically speaking, they constitute the same species [26].

### Comparative genomics and synteny analysis

**Fig. 3:**
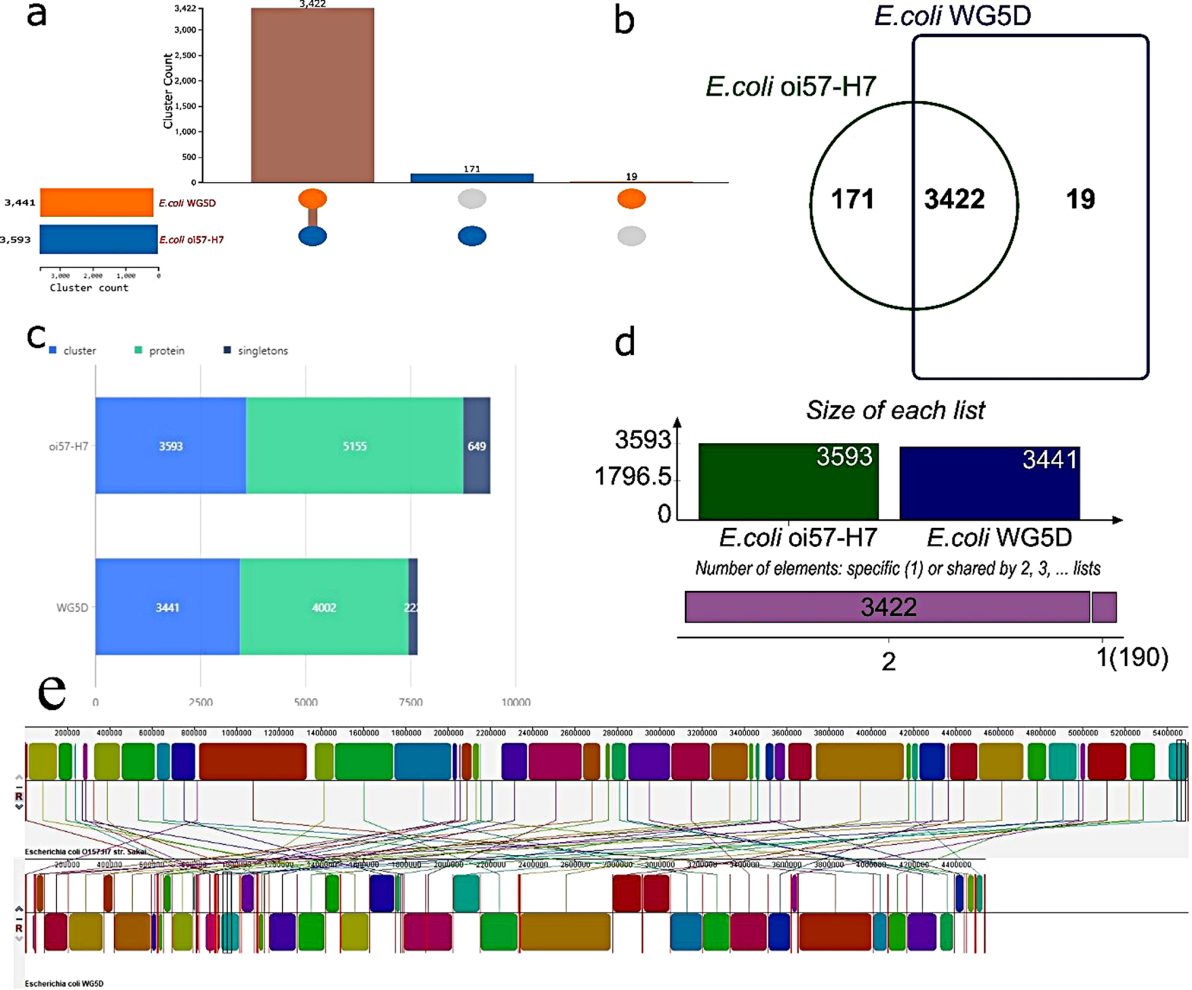
Comparison of *E. coli* WG5D genome sequence against *E. coli* representative genome sequence. (**a**) comparison of protein-coding genes in the genomes (**b**) Venn diagram showing the number of shared and unique clusters of orthologous genes (**c**) numbers of protein clusters and singletons in each genome (**d**) genome size comparison (**e**) Synteny analysis of the strains WG5D and oi57:H7 genomes, pairwise alignments of genomes were generated using Mauve. Boxes with same color indicate syntenic regions. Boxes below the horizontal strain line indicate inverted regions. Rearrangements are shown with colored lines. The scale is in nucleotides

Whole genome sequences of the *E. coli* WG5D and the *E. coli* representative strain (*E. coli* oi57:H7) were compared to identify specific genes and shared genes (Fig. 3a-d). There were 3422 shared genes between the two genomes. These orthologous protein-coding genes were relatively conserved in these two genomes. Additionally, *E. coli* WG5D has only 19 unique genes compared to the 171 unique genes in the reference strain (Fig. 3a-b). Furthermore, *E. coli* oi57:H7 has more clusters (3593), more proteins (5155), and more singletons (649) than WG5D, which has 3441 clusters, 4002 proteins, and 222 singletons (Fig. 3c). This is also confirmed in the size of the genomes depicted in Fig. 3d.

To further estimate the evolutionary distance between *E. coli* WG5D and the reference strain *E. coli* oi57:H7, their whole genome sequences were compared using Mauve (Fig. 3e). The alignments between *E. coli* WG5D and *E. coli* oi57:H7 showed that *E. coli* WG5D has a shorter chromosome length when compared to the reference. This result supports the comparative analysis result in Fig. 3a-d. Furthermore, several gene inversions and a large deletion region were detectable in *E. coli* WG5D, which were not present in *E. coli* oi57:H7. These results show that large local collinear block inversions occurred between *E. coli* WG5D and *E. coli* oi57:H7 (Fig. 3e).

### Genome mining for secondary metabolites

**Fig. 4:**
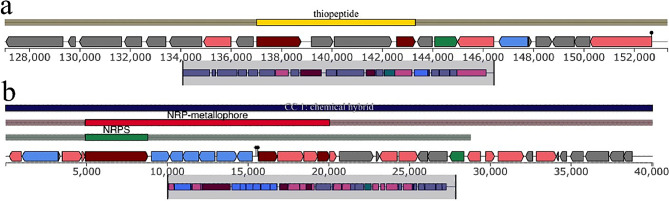
Secondary metabolites predicted by antismash

Bacterial whole genome sequencing data has improved the use of biosynthetic gene clusters (BGC) of secondary metabolite antimicrobial compounds in the discovery of antimicrobial natural products. *E. coli* WG5D genome revealed the presence of two BGC regions encoding for antimicrobial compounds of the types thiopeptides and NRPs proteins (Fig. 4). The thiopeptides include the *YcaO* and *Fer4_12* proteins. In contrast, the NRP proteins identified by antismash include NRPs region related to enterobactin siderophore biosynthesis.

### Mining for strain serotype, pathogenicity, MGEs, and virulence factors

**Fig. 5:**
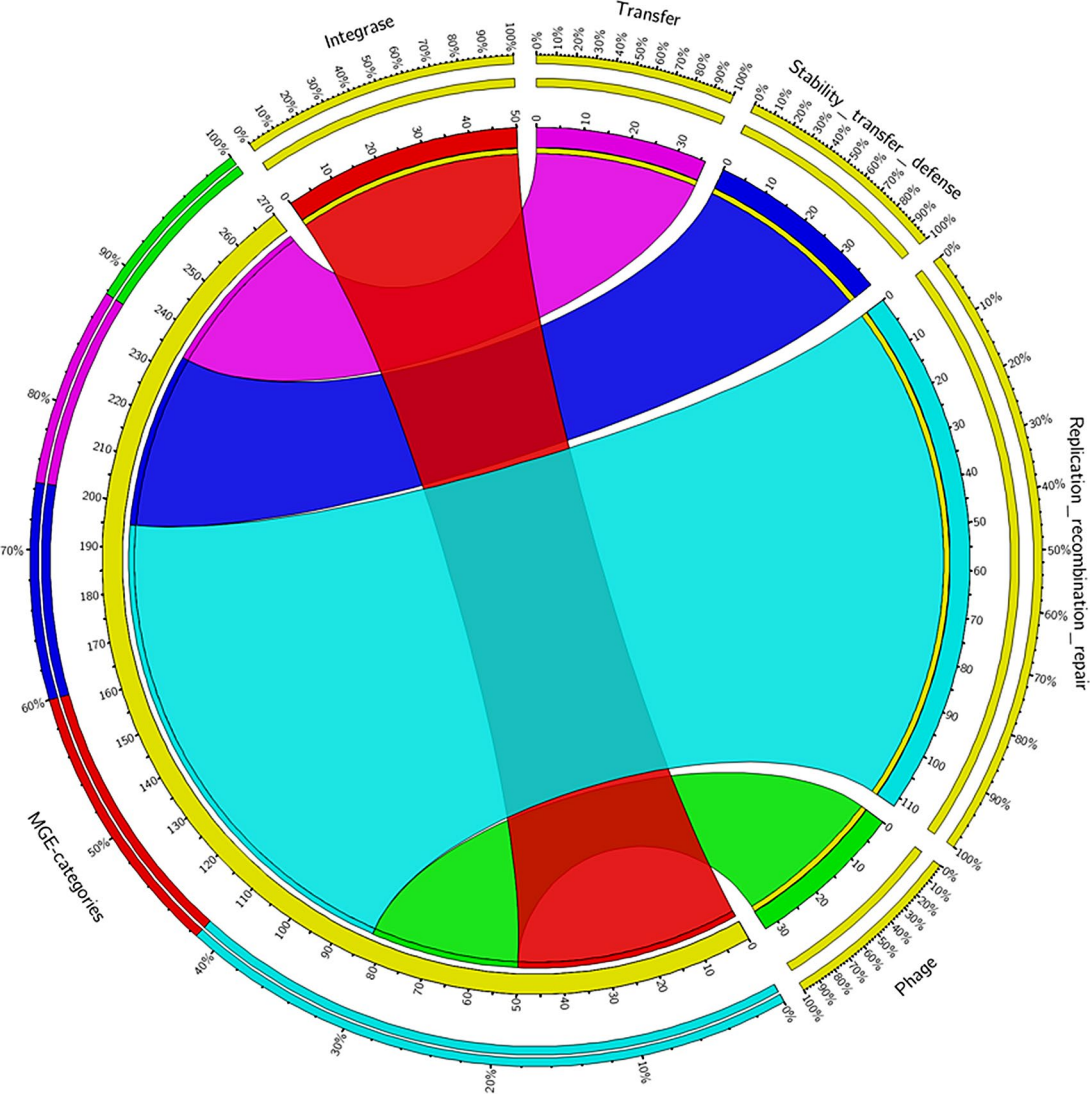
Circos plot showing the distribution of the identified MGEs categories in the WG5D genome

WG5D was identified as an H19 serotype (Table S4) and predicted to be a human pathogen with a probability rate of 93.2% (Table S5). It should be noted that the strain has the potential to cause infection, but it is not proven. A total of 270 MGEs categorized into 5 groups based on their functions (Fig. 5, Table S6) were further identified. MGE elements constitute replication, recombination, and repair functions making the largest number with a total of 112 elements, while phage elements are the least with a total of 33 identified (Fig. 5). Other identified elements include those of stability, transfer, and integrase.

A total of 15 virulence factors were predicted, including those represented in heat stress (*clpK1*), motility (*fimH, yehABCD*), adhesion (*fdeC*), haemolysis (*hlyE*), and tellurium ion resistance (*terC*) (Table 1).

**Table 1:** virulence factors identified

Virulence factor	Identity	Protein function
AslA	94,09	-
clpK1	99,9	heat shock survival AAA family ATPase ClpK. thermal stress survival
csgA	100	curlin major subunit CsgA
fdeC	93,07	intimin-like adhesin FdeC
fimH	100	Type 1 fimbriae
gad	100	Glutamate decarboxylase
gad	99,93	Glutamate decarboxylase
hlyE	100	Avian E. coli haemolysin
nlpI	99,77	lipoprotein NlpI precursor
terC	100	Tellurium ion resistance protein
terC	99,9	Tellurium ion resistance protein
yehA	97,97	Outer membrane lipoprotein, YHD fimbriael cluster
yehB	97,82	Usher, YHD fimbriael cluster
yehC	97,63	Chaperone, YHD fimbriael cluster
yehD	98,53	Major pilin subunit, YHD fimbriael cluster

### Genome mining for antimicrobial resistance genes

RGI analysis revealed 49 antimicrobial resistance genes with 21 perfect hits and 28 strict hits (Table 2). These genes were predicted to have > 38% identity to well-characterized ARGs in the CARD database. Identified genes include those for multidrug resistance (*AcrFAESBR, baeRS, H-NS, mdtEFPONM, gadX, AcrA, cpxA, marA, evgS, leuO, KpnEF, rsmA, CRP, soxSR, MarR*), nitroimidazole (*msbA*), tetracycline (*emrKY*), peptide (*pmrF, bacA, eptA*), fluoroquinolone (*mdtH, emrAB, gyrA*), phosphonic acid (*mdtG*), aminoglycoside (*acrD, kdpF*), aminocoumarin ( *mdtCA*), cephalosporin ( *EC-15*), and glycopeptide (*vanG*).

**Table 2:** Antibiotic-resistant genes based on RGI analysis on CARD database

RGI Criteria	ARO Term	Detection Criteria	AMR Gene Family	Drug Class	Resistance Mechanism	% Identity of Matching Region	% Length of Reference Sequence
Perfect	AcrE	protein homolog model	resistance-nodulation-cell division (RND) antibiotic efflux pump	fluoroquinolone antibiotic, cephalosporin, cephamycin, penam	antibiotic efflux	100	100
Perfect	TolC	protein homolog model	ATP-binding cassette (ABC) antibiotic efflux pump, major facilitator superfamily (MFS) antibiotic efflux pump, resistance-nodulation-cell division (RND) antibiotic efflux pump	macrolide antibiotic, fluoroquinolone antibiotic, aminoglycoside antibiotic, carbapenem, cephalosporin, glycylcycline, cephamycin, penam, tetracycline antibiotic, peptide antibiotic, aminocoumarin antibiotic, rifamycin antibiotic, phenicol antibiotic, penem, disinfecting agents and antiseptics	antibiotic efflux	100	99,6
Perfect	msbA	protein homolog model	ATP-binding cassette (ABC) antibiotic efflux pump	nitroimidazole antibiotic	antibiotic efflux	100	100
Perfect	baeR	protein homolog model	resistance-nodulation-cell division (RND) antibiotic efflux pump	aminoglycoside antibiotic, aminocoumarin antibiotic	antibiotic efflux	100	100
Perfect	baeS	protein homolog model	resistance-nodulation-cell division (RND) antibiotic efflux pump	aminoglycoside antibiotic, aminocoumarin antibiotic	antibiotic efflux	100	100
Perfect	evgA	protein homolog model	major facilitator superfamily (MFS) antibiotic efflux pump, resistance-nodulation-cell division (RND) antibiotic efflux pump	macrolide antibiotic, fluoroquinolone antibiotic, penam, tetracycline antibiotic	antibiotic efflux	100	100
Perfect	emrK	protein homolog model	major facilitator superfamily (MFS) antibiotic efflux pump	tetracycline antibiotic	antibiotic efflux	100	110,26
Perfect	PmrF	protein homolog model	pmr phosphoethanolamine transferase	peptide antibiotic	antibiotic target alteration	100	100
Perfect	H-NS	protein homolog model	major facilitator superfamily (MFS) antibiotic efflux pump, resistance-nodulation-cell division (RND) antibiotic efflux pump	macrolide antibiotic, fluoroquinolone antibiotic, cephalosporin, cephamycin, penam, tetracycline antibiotic	antibiotic efflux	100	100
Perfect	mdtH	protein homolog model	major facilitator superfamily (MFS) antibiotic efflux pump	fluoroquinolone antibiotic	antibiotic efflux	100	100
Perfect	mdtG	protein homolog model	major facilitator superfamily (MFS) antibiotic efflux pump	phosphonic acid antibiotic	antibiotic efflux	100	100
Perfect	acrD	protein homolog model	resistance-nodulation-cell division (RND) antibiotic efflux pump	aminoglycoside antibiotic	antibiotic efflux	100	100
Perfect	mdtE	protein homolog model	resistance-nodulation-cell division (RND) antibiotic efflux pump	macrolide antibiotic, fluoroquinolone antibiotic, penam	antibiotic efflux	100	100
Perfect	mdtF	protein homolog model	resistance-nodulation-cell division (RND) antibiotic efflux pump	macrolide antibiotic, fluoroquinolone antibiotic, penam	antibiotic efflux	100	100
Perfect	gadX	protein homolog model	resistance-nodulation-cell division (RND) antibiotic efflux pump	macrolide antibiotic, fluoroquinolone antibiotic, penam	antibiotic efflux	100	100
Perfect	*Escherichia coli* acrA	protein homolog model	resistance-nodulation-cell division (RND) antibiotic efflux pump	fluoroquinolone antibiotic, cephalosporin, glycylcycline, penam, tetracycline antibiotic, rifamycin antibiotic, phenicol antibiotic, disinfecting agents and antiseptics	antibiotic efflux	100	100
Perfect	cpxA	protein homolog model	resistance-nodulation-cell division (RND) antibiotic efflux pump	aminoglycoside antibiotic, aminocoumarin antibiotic	antibiotic efflux	100	100
Perfect	kdpE	protein homolog model	kdpDE	aminoglycoside antibiotic	antibiotic efflux	100	100
Perfect	emrA	protein homolog model	major facilitator superfamily (MFS) antibiotic efflux pump	fluoroquinolone antibiotic	antibiotic efflux	100	100
Perfect	emrB	protein homolog model	major facilitator superfamily (MFS) antibiotic efflux pump	fluoroquinolone antibiotic	antibiotic efflux	100	100
Perfect	marA	protein homolog model	resistance-nodulation-cell division (RND) antibiotic efflux pump, General Bacterial Porin with reduced permeability to beta-lactams	fluoroquinolone antibiotic, monobactam, carbapenem, cephalosporin, glycylcycline, cephamycin, penam, tetracycline antibiotic, rifamycin antibiotic, phenicol antibiotic, penem, disinfecting agents and antiseptics	antibiotic efflux, reduced permeability to antibiotic	100	100
Strict	AcrF	protein homolog model	resistance-nodulation-cell division (RND) antibiotic efflux pump	fluoroquinolone antibiotic, cephalosporin, cephamycin, penam	antibiotic efflux	99,71	100
Strict	AcrS	protein homolog model	resistance-nodulation-cell division (RND) antibiotic efflux pump	fluoroquinolone antibiotic, cephalosporin, glycylcycline, cephamycin, penam, tetracycline antibiotic, rifamycin antibiotic, phenicol antibiotic, disinfecting agents and antiseptics	antibiotic efflux	99,55	100
Strict	bacA	protein homolog model	undecaprenyl pyrophosphate related proteins	peptide antibiotic	antibiotic target alteration	99,63	100
Strict	Escherichia coli mdfA	protein homolog model	major facilitator superfamily (MFS) antibiotic efflux pump	tetracycline antibiotic, disinfecting agents and antiseptics	antibiotic efflux	97,07	100
Strict	YojI	protein homolog model	ATP-binding cassette (ABC) antibiotic efflux pump	peptide antibiotic	antibiotic efflux	99,63	100
Strict	mdtC	protein homolog model	resistance-nodulation-cell division (RND) antibiotic efflux pump	aminocoumarin antibiotic	antibiotic efflux	99,51	201,56
Strict	mdtA	protein homolog model	resistance-nodulation-cell division (RND) antibiotic efflux pump	aminocoumarin antibiotic	antibiotic efflux	99,04	100
Strict	evgS	protein homolog model	major facilitator superfamily (MFS) antibiotic efflux pump, resistance-nodulation-cell division (RND) antibiotic efflux pump	macrolide antibiotic, fluoroquinolone antibiotic, penam, tetracycline antibiotic	antibiotic efflux	99,58	98,58
Strict	emrY	protein homolog model	major facilitator superfamily (MFS) antibiotic efflux pump	tetracycline antibiotic	antibiotic efflux	99,41	100
Strict	mdtP	protein homolog model	major facilitator superfamily (MFS) antibiotic efflux pump	nucleoside antibiotic, disinfecting agents and antiseptics	antibiotic efflux	97,95	100
Strict	mdtO	protein homolog model	major facilitator superfamily (MFS) antibiotic efflux pump	nucleoside antibiotic, disinfecting agents and antiseptics	antibiotic efflux	99,41	100
Strict	mdtN	protein homolog model	major facilitator superfamily (MFS) antibiotic efflux pump	nucleoside antibiotic, disinfecting agents and antiseptics	antibiotic efflux	99,71	100
Strict	eptA	protein homolog model	pmr phosphoethanolamine transferase	peptide antibiotic	antibiotic target alteration	99,63	100
Strict	leuO	protein homolog model	major facilitator superfamily (MFS) antibiotic efflux pump	nucleoside antibiotic, disinfecting agents and antiseptics	antibiotic efflux	99,04	100
Strict	EC-15	protein homolog model	EC beta-lactamase	Cephalosporin	antibiotic inactivation	98,41	100
Strict	*Klebsiella pneumoniae* KpnE	protein homolog model	small multidrug resistance (SMR) antibiotic efflux pump	macrolide antibiotic, aminoglycoside antibiotic, cephalosporin, tetracycline antibiotic, peptide antibiotic, rifamycin antibiotic, disinfecting agents and antiseptics	antibiotic efflux	82,2	100,83
Strict	*Klebsiella pneumoniae* KpnF	protein homolog model	small multidrug resistance (SMR) antibiotic efflux pump	macrolide antibiotic, aminoglycoside antibiotic, cephalosporin, tetracycline antibiotic, peptide antibiotic, rifamycin antibiotic, disinfecting agents and antiseptics	antibiotic efflux	84,4	100
Strict	acrB	protein homolog model	resistance-nodulation-cell division (RND) antibiotic efflux pump	fluoroquinolone antibiotic, cephalosporin, glycylcycline, penam, tetracycline antibiotic, rifamycin antibiotic, phenicol antibiotic, disinfecting agents and antiseptics	antibiotic efflux	99,9	100
Strict	vanG	protein homolog model	glycopeptide resistance gene cluster, Van ligase	glycopeptide antibiotic	antibiotic target alteration	38,23	104,3
Strict	mdtM	protein homolog model	major facilitator superfamily (MFS) antibiotic efflux pump	fluoroquinolone antibiotic, lincosamide antibiotic, nucleoside antibiotic, phenicol antibiotic, disinfecting agents and antiseptics	antibiotic efflux	97,8	100
Strict	rsmA	protein homolog model	resistance-nodulation-cell division (RND) antibiotic efflux pump	fluoroquinolone antibiotic, diaminopyrimidine antibiotic, phenicol antibiotic	antibiotic efflux	85,25	100
Strict	CRP	protein homolog model	resistance-nodulation-cell division (RND) antibiotic efflux pump	macrolide antibiotic, fluoroquinolone antibiotic, penam	antibiotic efflux	99,52	100
Strict	*Escherichia coli* gyrA conferring resistance to fluoroquinolones	protein variant model	fluoroquinolone resistant gyrA	fluoroquinolone antibiotic	antibiotic target alteration	99,77	100
Strict	*Haemophilus influenzae* PBP3 conferring resistance to beta-lactam antibiotics	protein variant model	Penicillin-binding protein mutations conferring resistance to beta-lactam antibiotics	cephalosporin, cephamycin, penam	antibiotic target alteration	53,11	96,39
Strict	*Escherichia coli* soxS with mutation conferring antibiotic resistance	protein overexpression model	ATP-binding cassette (ABC) antibiotic efflux pump, major facilitator superfamily (MFS) antibiotic efflux pump, resistance-nodulation-cell division (RND) antibiotic efflux pump, General Bacterial Porin with reduced permeability to beta-lactams	fluoroquinolone antibiotic, monobactam, carbapenem, cephalosporin, glycylcycline, cephamycin, penam, tetracycline antibiotic, rifamycin antibiotic, phenicol antibiotic, penem, disinfecting agents and antiseptics	antibiotic target alteration, antibiotic efflux, reduced permeability to antibiotic	100	100
Strict	*Escherichia coli* soxR with mutation conferring antibiotic resistance	protein overexpression model	ATP-binding cassette (ABC) antibiotic efflux pump, major facilitator superfamily (MFS) antibiotic efflux pump, resistance-nodulation-cell division (RND) antibiotic efflux pump	fluoroquinolone antibiotic, cephalosporin, glycylcycline, penam, tetracycline antibiotic, rifamycin antibiotic, phenicol antibiotic, disinfecting agents and antiseptics	antibiotic target alteration, antibiotic efflux	100	100
Strict	*Escherichia coli* AcrAB-TolC with AcrR mutation conferring resistance to ciprofloxacin, tetracycline, and ceftazidime	protein overexpression model	resistance-nodulation-cell division (RND) antibiotic efflux pump	fluoroquinolone antibiotic, cephalosporin, glycylcycline, penam, tetracycline antibiotic, rifamycin antibiotic, phenicol antibiotic, disinfecting agents and antiseptics	antibiotic target alteration, antibiotic efflux	100	100
Strict	*Escherichia coli* AcrAB-TolC with MarR mutations conferring resistance to ciprofloxacin and tetracycline	protein overexpression model	resistance-nodulation-cell division (RND) antibiotic efflux pump	fluoroquinolone antibiotic, cephalosporin, glycylcycline, penam, tetracycline antibiotic, rifamycin antibiotic, phenicol antibiotic, disinfecting agents and antiseptics	antibiotic target alteration, antibiotic efflux	100	100

## Discussion

Water treatment plants are designed to treat water from various sources exposed to various contaminants. Animal wastes, municipal wastes, sewage, etc., might be a source of contaminant exposure in these water sources. The presence of these contaminants has been associated with the development of antibiotic-resistant bacteria and ARGs in the eventual effluent of water treatment plants. Commensal strains may take up antibiotics from animal wastes. These can lead to the development of antibiotic-resistant genes in these strains to fight for survival, which may increase public health risks. Hence, evaluation and monitoring of ARGs is important in preventing the transfer of ARGs. Various genetic elements, including genomic islands, are important sources of the transfer of genes between species. Genomic islands (GIs) are specific regions of the prokaryotic genome that are associated with the acquisition of accessory genes through horizontal gene transfer (HGT) [27, 28]. These regions are typically absent from the genomes of nonpathogenic strains but present in pathogenic strains [27].

In this study, a comprehensive genomic analysis was carried out to uncover insights related to antimicrobial and virulence genes, pathogenicity, multi-drug efflux pumps, transporter genes, stress protection mechanisms, and more. Furthermore, the various genomic features in the test isolate were explored. The *E. coli WG5D* genome features many multidrug efflux transporters conferring antibiotic resistance. Similar findings was reported by X Shi, M Chen, Z Yu, JM Bell, H Wang, I Forrester, H Villarreal, J Jakana, D Du, BF Luisi, et al. [29]. Their study provides insights into this multi-drug efflux pumps in situ structure and assembly, highlighting its role in conferring antibiotic resistance. The AcrAB-TolC efflux pump comprises the outer membrane protein *TolC*, the periplasmic adaptor protein *AcrA*, and the inner membrane transporter *AcrB* from the resistance-nodulation-cell division (RND) superfamily. This directly supports the presence of multi-drug efflux transporters in *E. coli* and their role in antibiotic resistance. In the strain WG5D, we observed genes related to quorum-sensing signaling molecules, such as the LysR-family proteins. LysR-type regulators are recognized transcription factors governing the expression of numerous genes engaged in diverse biological roles. These encompass bacterial virulence, biofilm construction, quorum sensing (QS), and the response to different stresses, including oxidative and metal-based compounds. These cumulative impacts can potentially affect the organism’s vulnerability to antibiotics ultimately [30]. For example, EP O’Grady, DT Nguyen, L Weisskopf, L Eberl and PA Sokol [31] reported the suppression of *cepIR* and *cciIR* QS genes in *B. cenocepacia* by *ShvR*, ultimately affecting AHL activity. In addition, the suppression of QS might result in a reduction of biofilm matrices and a disruption of their capability to retain cells within the biofilm structure. This could subsequently heighten the sensitivity of these biofilms to antibiotics [32]. This study also identified metal transporters, secreting systems, flagellar biosynthesis and regulatory proteins, and other important survival genes after annotating the *E. coli WG5D* genome. Several studies have reported similar findings in other *E. coli* species. For example, in their study, P Kong, G Huang and W Liu [33] provide insights into identifying protein complexes and functional modules in *E. coli*, which may include metal transporters as part of the cellular machinery. In another study by H Sun, M Wang, Y Liu, P Wu, T Yao, W Yang, Q Yang, J Yan and B Yang [34], the regulatory mechanisms of flagellar motility and biosynthesis in enterohemorrhagic *E. coli* Oi57:H7 (EHEC Oi57:H7) was extensively studied, focusing on flagellar gene regulation by environmental factors, regulatory proteins, and small regulatory RNAs. Additionally, the stochastic transcriptional pulses that orchestrate flagellar biosynthesis in *E. coli* have been investigated, revealing a deterministic transcriptional program that governs flagellum biosynthesis [35]. These studies shed light on the intricate regulatory processes that control flagellar biosynthesis and motility in *E. coli*. Furthermore, metal transporters play crucial roles in metal homeostasis and resistance. For example, the yersiniabactin metallophore system in *E. coli* is involved in copper import, highlighting the importance of metal transport systems in bacterial physiology and adaptation to metal stress [36]. The regulatory landscape of *E. coli* is complex, involving a wide array of regulatory proteins, transcription factors, and molecular chaperones. Identifying protein complexes and functional modules in *E. coli* protein-protein interaction networks provides insights into the regulatory architecture of the bacterium, shedding light on the intricate regulatory networks that govern cellular processes [33].

An overview of shared syntenic genes between WG5D and its representative genome, *E. coli* Oi5:H7, are illustrated in Fig. 3e. There are fewer regions of synteny between the two genomes. These could be caused by HGT, gene shuffling, or de novo gene formation. Recent HGTs are expected to have high sequence identity with another species group from which it would have been transferred and not be found in the closely related species [37]. Therefore, these non-syntenic islands can be because of a mix of significant rearrangements, duplication events, and the emergence of specific genes. Conserved regions alongside extensively reorganized non-syntenic blocks suggest an evolutionary push for stability in certain regions, contrasted by frequent gene shuffling and rearrangements in other areas, referred to as rearrangement hotspots. Das Mitra et al. (2022) also reported the presence of synteny regions in *E. coli* genomes. They performed a comparative genomics analysis on different *E. coli* genomes and identified syntenic regions among the studied strains.

Furthermore, this genome analysis showed the presence of enterobactin siderophore. Enterobactin is important in *E. coli* for stress resistance. For example, K Casanova-Hampton, A Carey, S Kassam, A Garner, GL Donati, S Thangamani and S Subashchandrabose [38] provided evidence to support the roles of enterobactin in promoting *E. coli* survival during Cu stress.

Bacterial genomes show remarkable stability in the short term, but they possess a high degree of flexibility from an evolutionary perspective. This balance between genome stability and adaptability is vital for the survival and thriving of bacteria over time [39]. Interestingly, genomic rearrangements are not confined to different species but are also observed within members of the same bacterial species [40]. For instance, during a long-term evolution experiment using *E. coli*, 110 genomic rearrangements were identified, including 19 inversions [41]. Approximately 70% of these rearrangements were associated with recombination between insertion sequence (IS) elements [41]. MGEs likely play a crucial role in driving genome rearrangement dynamics in this bacterium. Furthermore, MGEs, like IS elements, play a pivotal role in bacterial evolution by facilitating genomic rearrangements and promoting the acquisition of new genes, which are instrumental for bacterial pathogens’ adaptive capabilities [42–44]. The pathogenic potential of *E. coli* WG5D was investigated through genome mining and comparative genomics. Previous research has suggested that bacterial strains with larger genomes tend to possess increased adaptability to complex environments due to their greater number of metabolism- and stress-tolerance-related genes [45, 46]. A diverse array of ARGs were discovered in the genome of *E. coli* WG5D. genes conferring resistance to tetracycline, cephalosporin, fluoroquinolones, aminoglycoside, glycopeptides etc., the presence of these multi-drug resistance genes in this strain is potentially worrisome for human health. These multi-drug resistant genes have been attributed to HGT [47]. Observation of multi-drug resistance genes in this strain aligns with the report of Q Li, W Chang, H Zhang, D Hu and X Wang [48], where they specifically discuss the presence of antibiotic resistance genes, including *bla*_*CTX−M−15*_, *bla*_*TEM−1*_, and *qnrS1*, in ESBLs-producing *E. coli* isolated from wastewater treatment plants. Their study further highlights the role of plasmids in the transfer of multiple antibiotic resistance in *E. coli*, providing direct evidence of the existence of multi-drug resistance genes in these bacterial species.

The comprehensive genomic analysis of *E. coli* WG5D presented in this study unveils critical insights with substantial implications for public health, particularly in drinking water safety. The presence of a diverse array of ARGs, including those conferring resistance to tetracycline, cephalosporin, fluoroquinolones, aminoglycoside, and glycopeptides, raises concerns about the potential dissemination of multidrug-resistant strains into water sources. Given that water treatment plants are designed to address various contaminants, including those from animal wastes and municipal sewage [49], the risk of ARGs persisting in the effluent poses challenges to public health. To address these concerns, it is imperative to implement robust monitoring strategies for antibiotic resistance in water sources. Continuous surveillance and analysis of water samples, especially those from treatment plants, can provide valuable data on the prevalence and dynamics of antibiotic-resistant bacteria. Additionally, the identification of genomic islands and mobile genetic elements in *E. coli* WG5D underscores the importance of understanding horizontal gene transfer mechanisms in water environments. Future research efforts should focus on elucidating the pathways through which antibiotic resistance spreads in water systems, allowing for the development of targeted interventions.

In light of these findings, public health interventions should prioritize the establishment of stringent monitoring protocols in water treatment facilities and the implementation of advanced molecular techniques for the early detection of emerging antibiotic resistance patterns. Furthermore, collaborative efforts between researchers, policymakers, and water management authorities are essential to formulate and implement effective strategies to mitigate the potential risks of antibiotic-resistant bacteria in water sources. This study serves as a foundation for shaping evidence-based policies to safeguard water quality and public health.

## Conclusions

The findings in this study have substantial implications for public health, especially in drinking water safety. The potential transmission of antibiotic-resistant strains through water sources underscores the importance of continued research and heightened surveillance to monitor and mitigate these risks. Future research endeavors should focus on elucidating the precise transmission mechanisms and assessing the broader ecological impact of such resistant strains. Additionally, identifying diverse antibiotic resistance genes emphasizes the urgency of developing robust resistance monitoring strategies and implementing effective interventions. This study serves as a foundational contribution to advancing our knowledge of microbial behavior and provides essential insights for shaping infection management strategies in the face of evolving antibiotic resistance challenges.

## Methods

### Isolation and genome sequencing

The WG5D strain was isolated from a drinking water distribution source in North-West Province, South Africa, in August 2016 following the method described in CC Bezuidenhout, LG Molale-Tom, RK Kritzinger and OS Olanrewaju [50] and RK Kritzinger, LG Molale-Tom, OS Olanrewaju and CC Bezuidenhout [51]. Detailed sampling strategy and study design have been reported by RK Kritzinger [52]. The water source from where this strain was isolated was collected from distribution water i.e. after treatment [52]. Strain isolation was performed on nutrient agar at 37 °C for 24 h. Single colonies were picked, streaked onto nutrient agar three consecutive times, and grown anaerobically for 24 h to obtain pure isolates [51]. The DNA was extracted using the chemagic DNA bacteria kit (PerkinElmer, Germany), following the manufacturer’s protocol. The gDNA was quantified by the NanoDrop-800 spectrophotometer (Thermo Fisher Scientific, Wilmington, NC, USA) and Qubit (ThermoFisher Scientific, US) following the manufacturer’s protocol [52]. Paired-end Illumina library was prepared using Nextera XT Library Preparation kit (Illumina, US) and sequenced for (2 × 300 bp) cycles on Illumina MiSeq [50, 52]. Accordingly, the DNA library was prepared using Nextera XT library (Illumina, San Diego, CA, USA) targeted for the genome with 1 ng genomic DNA following the manufacturer’s recommendations [53]. Briefly, target genomic DNA was simultaneously fragmented and then tagged with adapter sequences in a single step using Nextera transposome (Nextera XT DNA Library Preparation Kit, Illumina, San Diego, CA, USA) [50]. Tagmented DNA was then amplified using a limited-cycle (12-cycle) PCR program. To purify the library DNA, amplified DNA was cleaned with AMPure XP beads [51]. Thereafter, the Nextera library was quantified using Qubit, and the size profile was determined on Agilent Technology 2100 Bioanalyzer using a high-sensitivity DNA chip (Agilent Technologies, Waldbronn, Germany) [50, 51]. The library for sequencing was normalized to 1nM and pooled. Then, the 1nM pooled library was diluted and NaOH-denatured before loading for the sequencing run on a MiSeq sequencer (MiSeq reagent kit V2-300 cycles) [50, 51].

### Assembly and annotation

The raw paired-end fastq reads (2 × 300 bp) were quality-checked using FastQC v.0.11.7 [54] followed by trimming of low-quality bases using Trimmomatic v.0.39 [55] and quality-checked again using FastQC v.0.11.7. The cleaned reads were assembled using SPAdes v.3.15.5 [56]. To evaluate the quality of the genome assembly, Quast (v.5.0.2) [57] was used, and CheckM was used to assess completeness and contamination (v.1.1.6) [58]. Further genomic analysis, annotation, and other comparative genomics studies were carried out using this WG5D draft assembly. The assembled draft genome of isolate WG5D was annotated using the Rapid Annotation System Technology (RAST) Pipeline [59]. The genome and its typical features were visualized using Proksee (v 1.1.2) [60]. Genomic islands were predicted using IslandViewer 4 server [61]. Default parameters were used in all programs except where otherwise stated.

### Genome-based phylogenetic analysis

The genome sequence was uploaded to the Type (Strain) Genome Server (TYGS), a free bioinformatics platform available at https://tygs.dsmz.de, for a whole genome-based taxonomic analysis [23]. The analysis also used recently introduced methodological updates and features [62]. TYGS’s sister database provided information on nomenclature, synonymy, and associated taxonomic literature, the List of Prokaryotic names with Standing in Nomenclature (LPSN, available at https://lpsn.dsmz.de). The results were provided by the TYGS on 2023-07-17. The TYGS analysis was subdivided into the following steps:

### Determination of closely related type strains

Determination of the closest type strain genomes was done in two complementary ways: First, all user genomes were compared against all type strain genomes available in the TYGS database via the MASH algorithm, a fast approximation of intergenomic relatedness [63], and the ten type strains with the smallest MASH distances chosen per user genome. Second, an additional set of ten closely related type strains was determined via the 16S rDNA gene sequences. These were extracted from the user genomes using RNAmmer [64]. Each sequence was subsequently BLASTed [65] against the 16S rDNA gene sequence of each currently 19225 type strain available in the TYGS database. This was used as a proxy to find the best 50 matching type strains (according to the bit score) for each user genome and to subsequently calculate precise distances using the Genome BLAST Distance Phylogeny approach (GBDP) under the algorithm ‘coverage’ and distance formula d5 [66]. These distances were finally used to determine each user genome’s 10 closest type strain genomes.

### Pairwise comparison of genome sequences

All pairwise comparisons among the genomes were conducted using GBDP for the phylogenomic inference, and accurate intergenomic distances were inferred under the algorithm ‘trimming’ and distance formula d5 [66]. 100 distance replicates were calculated each. Digital DDH values and confidence intervals were calculated using the recommended settings of the GGDC 3.0 [62, 66].

### Phylogenetic inference

The resulting intergenomic distances were used to infer a balanced minimum evolution tree with branch support via FASTME 2.1.6.1, including the SPR postprocessing [67]. Branch support was inferred from 100 pseudo-bootstrap replicates each. The trees were rooted at the midpoint [68] and visualized with PhyD3 [69].

### Type-based species and subspecies clustering

The type-based species clustering using a 70% dDDH radius around each of the 10 type strains was done as previously described [23]. Subspecies clustering was done using a 79% dDDH threshold as previously introduced [70].

In addition, the in silico DDH value was calculated by the Genome-to-Genome distance calculator (GGDC) to compare the genome. The phylogenetic tree was constructed based on the average nucleotide identity (ANI). The overall similarity between the whole-genome sequences was calculated using fastANI [71].

### Analysis of genes Associated with antimicrobial resistance, virulence, and secondary metabolites

The genome of WG5D was mined for biosynthetic gene clusters of antimicrobial compounds, including NRPs, PKs, NRPs-PKs hybrids, bacteriocins, and terpenes, with RAST system [59], antiSMASH (v 6.0) [72]. Annotated protein-coding sequences of *E. coli* WG5D were further aligned against the carbohydrate-active enzyme (CAZy) database using dbCAN2 with the threshold of E-value1e-15 [73]. Phage annotation was performed using the PHAge Search Tool with Enhanced Sequence Translation (PHASTEST) web server [74]. Web tools (www.genomicepidemiology.org) were used for the determination of strain serotype [75], pathogenicity [76], and VirulenceFinder [77] for the detection of *E. coli* virulence genes. The virulence genes were viewed using circos [78]. Mobile genetic elements (MGEs) were identified using the mobileOG-db software (v1.6) [79] and visualized using circus [78]. Antimicrobial resistance genes were mined using the Resistance Gene Identifier (RGI) tool of the Comprehensive Antibiotic Resistance Database (CARD) [80] using contigs file with the parameters “Perfect and Strict hits only” and “High quality/coverage”. Default settings were used in all analyses except where otherwise stated.

### Electronic supplementary material

Below is the link to the electronic supplementary material.

Supplementary Material 1

Supplementary Material 2

## Data Availability

This published article and its supplementary information files include all data generated or analyzed during this study. This Whole Genome Shotgun project has been deposited at DDBJ/ENA/GenBank under the accession JAUOOV000000000. The version described in this paper is version JAUOOV010000000. The BioProject accession number associated with this genome is PRJNA997104.
