# Supplementary figures and images for: Genome mining of Escherichia coli WG5D from drinking water source: unraveling antibiotic resistance genes, virulence factors, and pathogenicity
Source: BMC Genomics. 2024 Mar 8;25:263. doi: 10.1186/s12864-024-10110-x (PMC10924361; doi:10.1186/s12864-024-10110-x)

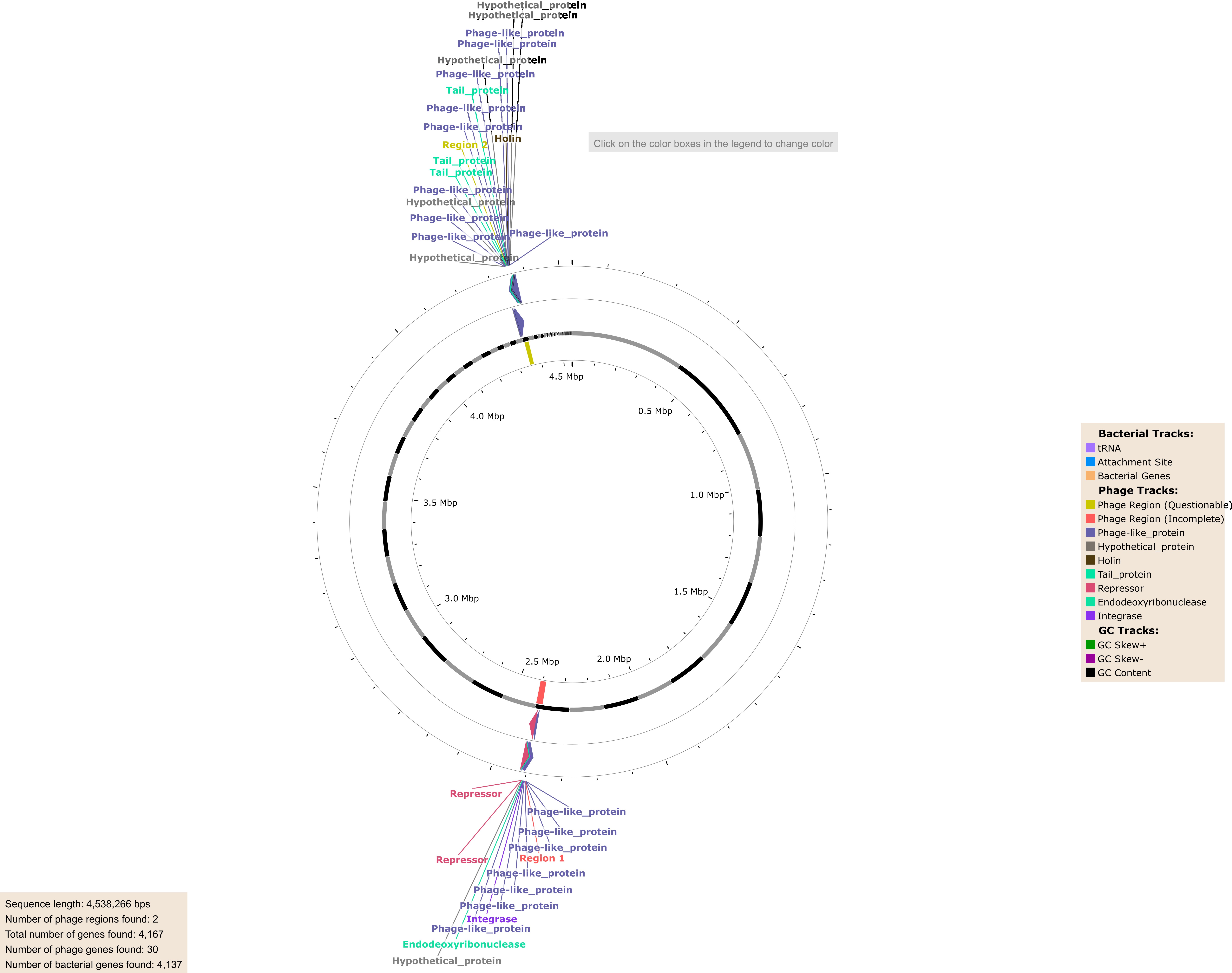

Supplement: Supplementary file 2 — Supplementary Material 2 [file 12864_2024_10110_MOESM2_ESM.jpg]
